# Health at Home

**Published:** 1873-06

**Authors:** 


### HALL'S

### JOURNAL OF HEALTH.

VOL. XX.—JUNE, 1873.—No. VI.

### "HEALTH AT HOME."

Twenty years ago the first number of Hall's Journal of Health was issued, without a single subscriber, and with the assurance of experienced publishers that it "never would succeed," because the object in view was the general good, and the great public would not appreciate it.

The first aim was to show the people how to keep well; advising that if any one was sick the wisest course was a prompt application to an educated physician. These have been the teachings of the Journal ever since. At that time it seemed to the great multitude among us to be a new idea, or rather, the idea does not appear to have entered the imagination of the people that there was anything else for the doctor to do but to attend to the sick and try and cure them as soon as possible.

A few years later a book was written in England on "Preventible Diseases," showing that a very large share of ordinary sickness could be avoided, and now "sanitary science" has become one of the leading studies of the times, and has attained such headway that there is no danger of its coming to a halt; so the new book takes another step in advance, aiming to show how every child born into the world may have a sound constitution and high moral sentiments, to live in health, comfort, and usefulness to the age of a hundred years.

It aims to show that honorable marriage and child-bearing are direct promoters of longevity; that the matron outlives the maid; the benedict, the bachelor; and that these results are uniform in all civilized communities.

It advocates the idea that deliberate efforts for the limitation of children is a great physical injury and moral wrong; that child-bearing never could have been intended to imperil life, and may in all cases become safe, and almost, if not entirely, painless, simply by attending mainly to what is eaten and drunken, with a reasonable care as to air, exercise, and cleanliness, and a rational attention to the state of the mind, heart, and temperaments during the full period of gestation.

The book advocates the idea that instead of half of all the children born dying before the seventh year, nine-tenths of them should reach old age. It shows, by examples given, that a large share of infant mortality, sickness, and suffering is directly traceable to improper dressing, to irregular feeding, and to unwise exposures; and to make it all plain, clear instructions are given for the management of children from the minute after birth until marriage; how to clothe them; how and when and on what to feed them, so that they shall never fret or cry; and how to manage their sleep that it shall be always good and never interfere materially with the mother's rest. To have a baby born well, and that shall never cry or keep its mother from sleeping, is worth something. Multitudes of nursing mothers seem almost

DRAGGED TO DEATH

with the sickness and crossness by day, and restlessness by night, of young children, all of which could be rectified in a week, more or less, completely, by regulating the eating properly and arranging suitable hours of sleep, as detailed in the book. Almost all the

DYSPEPTIAS AND CONSUMPTIONS

of mature life, among women, such as are most incurable, have their foundations laid in the teens of girlhood by bad habits of eating mainly; and in other part, for the want of knowledge in a few personal matters; the book gives full information as to all these points. Most of our

DAUGHTERS

are allowed to grow up in almost entire ignorance of housekeeping duties and industries, as sewing, patching, cutting, fitting, and making garments of all kinds; how to lay in supplies for the table, and managing household matters; how these things are to be learned and done so as to make a husband better satisfied at home than at any other place, are pointed out. It is the want of tidy, happy homes which drives multitudes of worthy men to clubs and drinking places; and mothers are made to understand that their daughters should be taught how to keep a husband as well as how to get one. Every family is liable to sudden and severe

SICKNESS AND ACCIDENT, and just as likely as not these come at midnight or in the depth of winter, or during fearful storms, and delay is death. The book details the means of meeting these things with domestic remedies, such as are found in every family, which, while they cure in many cases, save time and mitigate in all until the doctor comes; it shows how a hundred severe ailments can be absolutely and triumphantly cured by the proper use of hot water or a little grease or salt and mustard!

But for the benefit of those who are in out-of-the-way places, or who have not the means to employ physicians, over one thousand medical prescriptions are given from the books of the very best old-school physicians within the last fifty years. In this respect the book is extremely valuable to all who give medicine. But as there are advantages in

THE WATER-CURE TREATMENT, all forms of baths are minutely described for the benefit of those who prefer not to use drugs. The book aims also to enable the reader to know what is the matter with him, by giving him the prominent and distinguishing or peculiar symptoms of a given disease. There is one point on which almost every critic hurls his indignant darts—the want of system. The author did not want any system about it. His aim was to make a readable book, so that it might be turned at any part, and in a page or two one malady would be treated, then another wholly different. If forty consecutive pages are given to the half hundred different kinds of head-ache, it would aggravate the malady to have to read them all and then decide which of them was present in the case in hand. But if a man has sick head-ache, he can turn to the index and find out all about it; and that is what he wants; he feels no possible interest in any other kind. Every one who takes up a newspaper wants to read one thing and another, and not a dozen columns about one thing. The book was written to enable every one to read it and know what it means without any mental effort. That is the kind of reading we like ourselves, and judged that our human nature was the human nature of other people. May be, that is the reason why the people have bought and read over a hundred and fifty thousand copies of our different books. Some of them contain seven or eight hundred pages and more.

Another point in "Health at Home" is made very plain—that the state of the mother's mind during gestation shapes the mental and moral temperament of the child. This is of immeasurable importance, if true, because it puts it in the power of the mother to make her child an angel or an apollyon—seraphic or satanic.

The state of the mind, a few minutes before the child is nursed, fed from the mother's bosom, can cause convulsions and death within the hour; the facts are given; hence, when the child feeds from the natural fountain, the state of the mother's mind at the time, and for some period before, ought to be tranquil. What lessons these, for every manly husband to make it his study, at any sacrifice of ease or money, to render his wife as happy as possible, to do all he can to avert everything calculated to ruffle, to dispirit, to annoy; and more, to do all that is possible to bring prospective sunshine about her by hearty smiles, tenderness and warm sympathies, so as to impart strength and encouragement and hope. How little do men understand these things! and yet upon them do the destinies of their children depend to a very great extent.

Divorces are considered at length, the ground being taken that a large share of them is from a private cause on the part of the wife, although not suspected by her as the reason; but what it is, how it operates, and its very natural results are clearly pointed out; and as it is often a mere inadvertence, the result of ignorance or inconsideration, and moreover can be easily avoided, it is believed that this item alone will prevent many a woman from wrecking her domestic happiness and ruining the reputation of her family.

DIVORCE

is always disgraceful, and more, it is dreadful to both parties; the great public does not care to inquire who is in error; it takes the shorter course, and exclaims with contemptuous indifference, "O! no doubt one is as much to blame as the other," and both parties are set down as low-bred and vulgar people, who have no family pride and no proper sense of public shame; the result is, they are both gradually dropped from the visiting list, and are allowed to pass into disgraceful obscurity. But that cannot be the end of it; unavailing remorses corrode the heart, eat out the affections, and embitter the whole nature, making of life a lengthened misery and torture; these are results as to husband and wife, while to the innocent children, especially if they are girls, it is a ceaseless mortification, and their sensitive minds are daily wrung with agonies almost unendurable.

"Health at Home" was intended to be different from all other "Family Doctors," in going farther back than any of them, and preventing the birth of sickly and deformed children, making it as sure as in the animal creation. The method, not only possible, but practicable, is proposed, by which it can be made a reasonable certainty, that in any given case, the child that is to be born shall come into the world at its full and proper time, with a perfect physical form, a good constitution, and with high moral sentiments and temperament. The thoughtful reader will see at once the possibility of this by considering a few official facts. In any company of idiotic children it will be found that three-fourths of them are from parents, one or both of whom were frequently under the influence of spirituous liquors—that is, were habitual "drinkers." This has been already stated; surely, then, good characteristics of body and mind and heart will as certainly follow right living. But there is perhaps not one mother in a million who has ever made an intelligent effort to mould the character of her child by a rational attention to her own physical and mental state from the moment of impregnation until weaning. Surely the subject merits the thoughtful consideration of every educated mind.

